# Double plating with autogenous bone grafting as a salvage procedure for recalcitrant humeral shaft nonunion

**DOI:** 10.1186/s12891-020-03743-y

**Published:** 2020-11-21

**Authors:** Dongxu Feng, Xiaolong Wang, Liang Sun, Xiao Cai, Kun Zhang, Zhan Wang, Yangjun Zhu

**Affiliations:** Department of Orthopaedic Trauma, Hong Hui Hospital, Xi’an Jiaotong University School of Medicine, Xi’an, 710054 Shaanxi China

**Keywords:** Humerus, Recalcitrant nonunion, Plate, Bone graft

## Abstract

**Background:**

Although most cases of humeral shaft nonunion respond well to surgical intervention, surgeons still encounter patients with humeral shaft nonunion who have already undergone repeated surgeries for nonunion. This study retrospectively analyzed the efficacy of double locking compression plate (LCP) fixation in combination with autogenous iliac crest bone grafting for recalcitrant humeral shaft nonunion.

**Methods:**

A consecutive series of patients with aseptic recalcitrant humeral shaft nonunion underwent surgical treatment between May 2010 and August 2017 in our institution. Standardized treatment included thorough debridement, double LCP and screw fixation, and autogenous iliac bone grafting. The injury type and the duration of nonunion were recorded for all patients. The main outcome measurements were the Constant and Murley scale for shoulder function, Mayo elbow performance index (MEPI) for elbow function, and visual analog scale (VAS) for pain. In addition, all complications were documented.

**Results:**

The study cohort comprised six females and nine males with a mean age of 45.3 ± 13.1 years. Each patient had already undergone at least one failed surgery for humeral shaft nonunion. The average duration of nonunion before the index intervention was 126.8 ± 124.2 months. All patients achieved bone union without implant failure. At final follow-up, the mean Constant and Murley score and mean MEPI were significantly improved, and the mean VAS score was significantly decreased. Each patient was very satisfied with the treatment. Four patients had complications, including one with a superficial wound infection, one with radial nerve palsy, one with ulnar nerve palsy, and one with discomfort at the iliac crest.

**Conclusion:**

Double plate fixation combined with autogenous iliac crest bone grafting results in successful salvage of humeral shaft nonunion after prior failed surgical interventions.

## Background

Humeral diaphyseal fractures account for approximately 3 to 5% of all fractures and 30% of all humeral fractures, while 64% of humeral diaphyseal fractures involve the midshaft [1, 2].

The methods used to treat primary humeral diaphyseal fracture include conservative treatment, open reduction and internal fixation, closed reduction and intramedullary nailing is the standard treatment for midshaft fracture. Although these strategies can lead to a high healing rate with a good functional outcome, posttraumatic nonunion of the humeral shaft is uncommon [2]. The reported incidence of humeral shaft nonunion is 2–10% after non-surgical treatments and up to 13% after operative management; atrophic nonunion is the most common type, and is commonly seen in the midshaft region [3–5].

The development of humeral shaft nonunion is related to many factors, such as comminuted fracture, inadequate reduction and unstable fixation, poor blood supply of the soft tissue envelope, fixation with distraction, systemic state of the patient (especially comorbidities like diabetes or malnutrition), infection, age, smoking, and premature weight-bearing [6, 7]. Nonunion often has a multifactorial origin [8].

As the affected upper extremity often presents with pain and loss of function, patients with humeral shaft nonunion often need revisions to improve their life quality. Many surgical techniques for the treatment of humeral shaft nonunion have been described, such as open reduction and internal fixation with a locking compression plate (LCP) and bone graft, double plate fixation, allogeneic or autologous cortical bone grafting, intramedullary nailing, Ilizarov external fixation, and the addition of biologic augmentation or low-intensity pulsed ultrasound [9–11]. However, most authors report that the standard procedure for nonunion of the humeral shaft is open reduction, plating, and bone grafting [3–5, 7, 10]. It is particularly complicated and challenging to treat patients with recalcitrant nonunion who have already undergone at least one failed surgical treatment for nonunion, due to osteopenia, deformity, bone loss, soft tissue scarring, scalloping around the screws, and metallosis at the nonunion site [12]. Although many studies have reported the successful treatment of primary humeral diaphysis nonunion, few studies have specifically evaluated revision procedures for the salvage of persistent nonunion following failed initial nonunion interventions [2, 8]. As our previous study showed that double plate fixation combined with structural autologous iliac bone grafting results in reasonable treatment outcomes for limb nonunion [13], the aim of the present study was to evaluate the clinical outcomes of this treatment strategy for recalcitrant humeral shaft nonunion.

## Methods

### Study design and patients

This was a retrospective study of the medical records and radiographs of 15 consecutive patients who underwent an intervention for aseptic recalcitrant humeral shaft nonunion between May 2010 and August 2017 in the department of orthopedic trauma, Honghui Hospital, Xi’an Jiaotong University School of Medicine, Xi’an, China (Table 1). The inclusion criteria were: 1) patients who received one or more surgical revisions for nonunion of the humeral shaft at least 9 months ago, and the fracture had shown no visible progressive signs of healing for 3 months [14]; 2) pain and dysfunction requiring intervention; 3) a revision procedure comprising double plate fixation in combination with autogenous iliac crest bone grafting. The exclusion criteria were single plate fixation, internal nailing, external fixation, or infected nonunion.

**Table 1:** Demographic data

Patient	Gender	Side	Cause of injury	Type of primary injury	Site(thirds)	Type of nonunion	Time since injury(months)	Prior treatments	comorbidity
1	F	L	Tumbling	Closed fracture	Middle	Atrophic	120	Plate; plate+bone graft	None
2	M	L	Tumbling	Closed fracture	Middle	Synovial pseudarthrosis	368	Plate; plate+bone graft	Arrhythmia
3	M	L	Traffic accident	Closed fracture	Middle	Hypertrophic	259	Plate; plate+bone graft	Diabetes mellitus
4	F	L	Tumbling	Closed fracture	Middle	Atrophic	24	Splint; plate	None
5	M	L	Crashing	Closed fracture	Middle	Synovial pseudarthrosis	226	Splint; plate+bone graft	None
6	F	R	Crashing	Closed fracture	Middle	Hypertrophic	35	Cast; plate; bone graft	None
7	M	R	Traffic accident	Closed fracture	Middle	Hypertrophic	158	Cast; plate; plate+bone graft	None
8	M	L	Crashing	Closed fracture	Middle	Atrophic	21	Plate; bone graft	None
9	F	L	Tumbling	Open fracture	Distal	Atrophic	17	Debridement; plate; bone Graft	None
10	M	L	Tumbling	Closed fracture	Middle	Atrophic	19	Plate; bone graft	None
11	M	L	Crashing	Closed fracture	Middle-Distal	Atrophic	23	Cast; plate+bone graft	None
12	F	L	Tumbling	Closed fracture	Middle	Oligotrophic	20	Splint; plate	None
13	M	R	Crashing	Open fracture	Middle	Hypertrophic	319	Debridement; Plate; plate+bone graft; plate	None
14	F	R	Sports injury	Closed fracture	Middle-Distal	Atrophic	25	Cast; plate+bone graft	None
15	M	L	Traffic accident	Closed fracture	Middle	Hypertrophic	27	Plate; bone graft	None

*M* male, *F* female

Classification of nonunion based on Weber-Cech classification

Patients’ demographic and clinical data were retrieved from the medical records prior to revision treatment. Laboratory test results including complete blood count, C-reactive protein concentration, and erythrocyte sedimentation rate were also assessed to rule out infection. Comorbidities were addressed.

This study was approved by the Ethics Committee of Hong Hui Hospital, Xi’ an Jiaotong University. Informed consent was acquired of every patient to publish their individual clinical details and accompanying images.

### Surgical technique

**Fig. 1:**
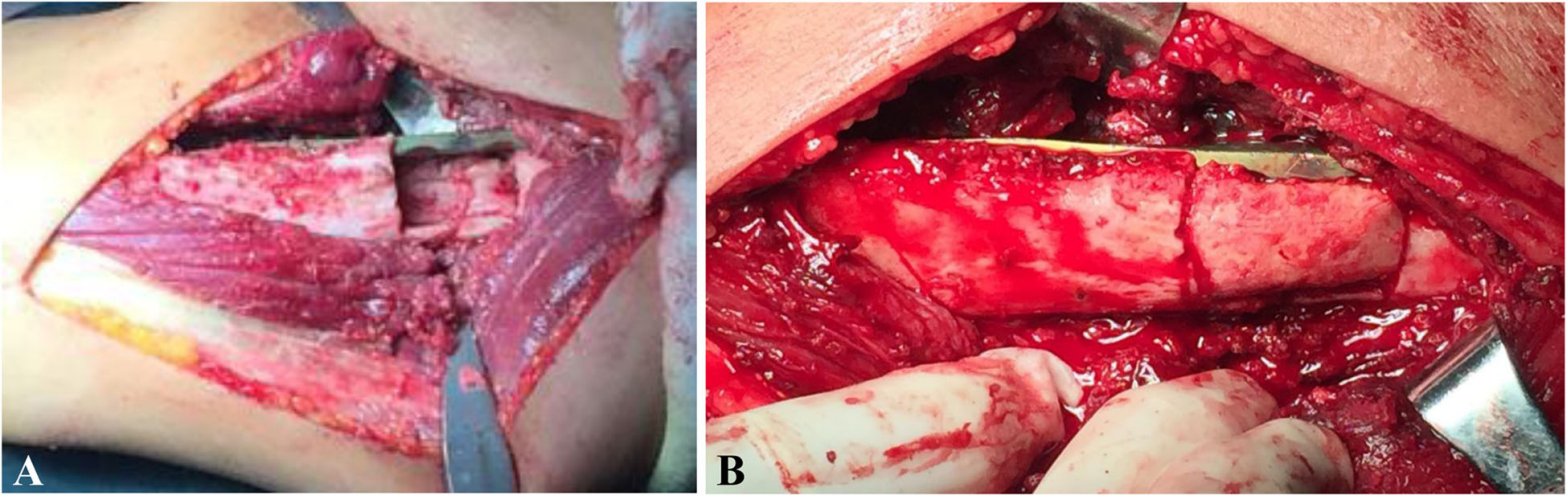
**a** A groove is made 90° perpendicular to the first plate at the side across the ends of the nonunion site. **b** A structural autologous bone graft is loaded into the bone groove

**Fig. 2:**
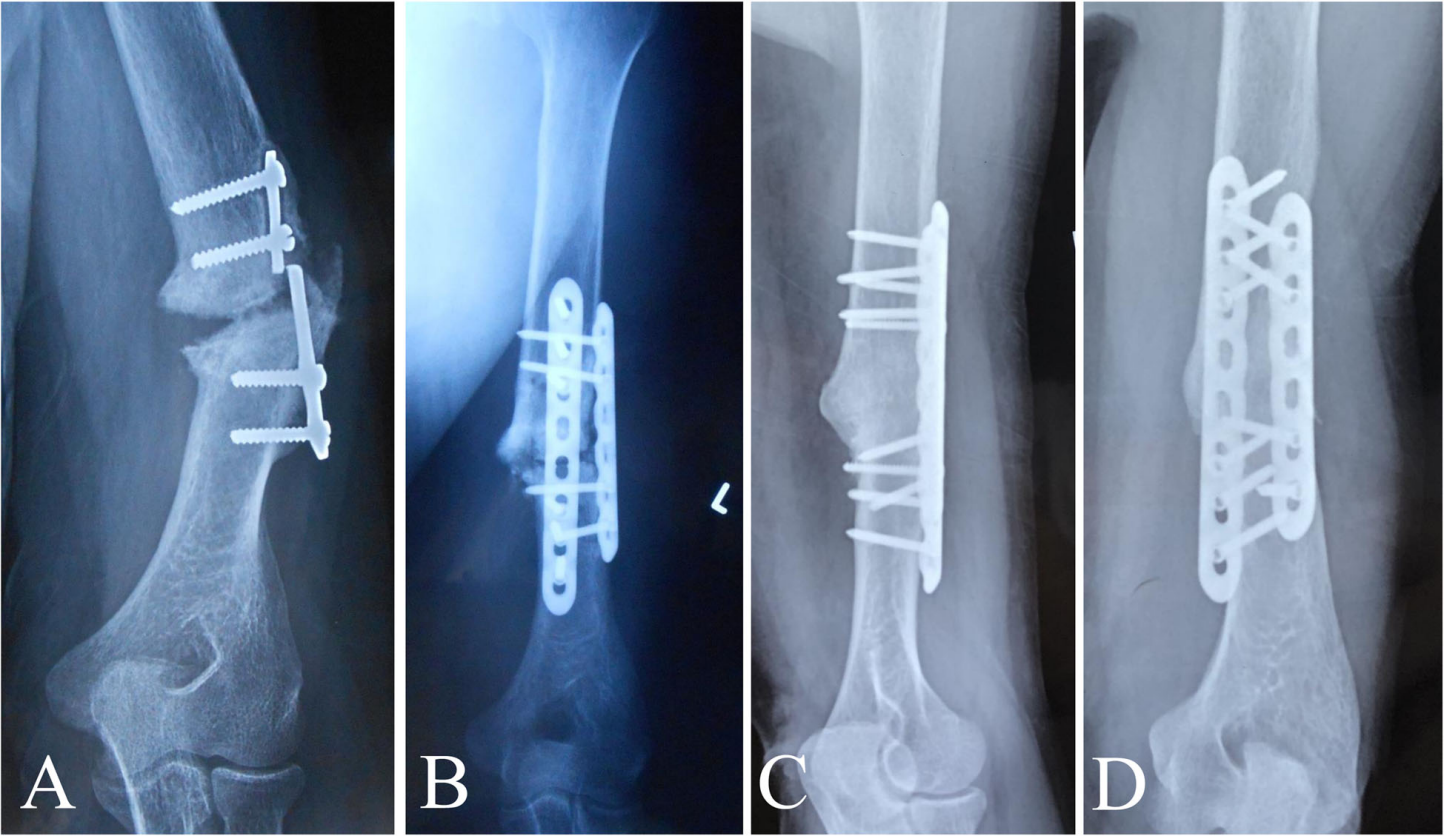
Images from a 58-year-old man who underwent plate fixation of a left humerus shaft fracture 30 years ago and was treated with plate fixation combined with autogenous iliac crest bone grafting because of nonunion 1 year postoperatively. However, the humeral fracture remained un-united until he visited our institution. **a** Preoperative plain radiograph showing classic synovial pseudarthrosis nonunion of the left humerus with a broken plate. **b** Radiograph taken immediately after revision surgery showing double locking compression plating with autogenous iliac crest bone grafting. **c**, **d** Radiographs showing consolidated bone union at 45 months after the index surgery

The surgical intervention was aimed at correcting deformities, maintaining bone alignment, and creating an environment conducive to bone healing. After the induction of general anesthesia, all operative procedures were performed by well-trained orthopedic surgeons. The surgical approach was dependent upon the previous surgical treatment, the nonunion site, and the surgeon’s preference. Generally, an anterolateral approach was used for nonunion in the proximal and middle thirds of the humerus, while a posterior approach was used for nonunion in the distal third [15]. During exposure, care was taken to identify and protect important structures, especially the radial nerve, as a thorough neurolysis was necessary due to the presence of abundant scar tissue from multiple surgeries. The fracture nonunion site was explored after the removal of the original failed fixation devices (except in two patients with no gap between the nonunion sites and stable plating with at least six layers of cortices fixed at each side, in whom we left the previous plate in place and added a second plate). A thorough debridement was then performed based on the Judet periosteal stripping technique [16], with the entire pseudocapsule, interposed fibrous tissue, and sclerotic bone excised until punctate bleeding was seen at the bony ends (Paprika sign) [5]. However, if there was any suspicion of low grade infection or septic pseudarthrosis during the debridement procedure, the treatment strategy was changed to external fixation. After opening the medullary canal, any angulation and rotation were corrected, and osteosynthesis was performed by using a 4.5-mm narrow LCP in compression mode to obtain cortex-to-cortex stability. A groove lying 90° perpendicular to the first plate was made at the side across the ends of the nonunion (Fig. 1a). An autogenous iliac crest bone graft was harvested, trimmed, and loaded into the bone groove, with the bone graft spanning the fracture (Fig. 1b). A second plate was then fixed to the front of the bone graft (Fig. 2). In addition, several pieces of cancellous bone were longitudinally packed to bridge the nonunion site. Samples were not routinely taken for microbiological testing. The wound was closed in layers.

### Postoperative management

Cephalosporin antibiotics were routinely given for 30 min preoperatively and continued for 24 h postoperatively, and drainage was left in place for 48 h. No external immobilization was prescribed; thus, supervised functional rehabilitation including gentle active and active-assisted range-of-motion exercises of the shoulder and elbow were begun on postoperative day 1. At 4 weeks postoperatively, aggressive range-of-motion exercises were initiated, while lifting of weights was not allowed until osseointegration or fracture healing was observed [7].

### Data collection and analysis

Postoperative follow-up including both clinical and radiographic evaluation was performed until the bone healed, and was then performed every 6 months by an independent observer. Osseous healing was defined as the presence of at least three of four healed cortices on plain radiography, and CT was not routinely performed unless it was difficult to judge healing on plain radiography [4]. The Mayo Elbow Performance Index (MEPI) was calculated preoperatively and at the most recent follow-up visit for each patient [17]. A MEPI score of 90–100 was considered an excellent result, 75–89 was considered good, 60–74 was considered fair, and less than 60 was considered poor. Shoulder function was evaluated using the Constant and Murley scale [18], with the result considered excellent if the score was 80–100, good if the score was 60–79, fair if the score was 40–59, and poor if the score was < 40. Pain was assessed using a visual analogue scale (VAS) from 0 to 10 [5]. Statistical analysis was performed using SPSS version 17.0 software (SPSS Inc., Chicago, IL). Differences in the findings were analyzed by paired-sample t-tests, and *P* < 0.05 was considered statistically significant.

## Results

The study cohort consisted of six women and nine men with a mean age of 45.3 ± 13.1 years (range, 23–62 years) (Table 2). The nonunion was in the left humerus in 11 patients and the right humerus in four. An anterolateral approach for revision was used in 12 patients, while a posterior approach was used in three. Based on the Weber-Cech classification [19], seven patients had atrophic nonunion, two had synovial pseudarthrosis, five were hypertrophic, and one was oligotrophic. Thirteen patients had closed fractures, while two had open fractures that eventually formed one atrophic nonunion and one hypertrophic nonunion at the time of the final intervention. All patients had aseptic nonunion. The mechanism of initial injury obtained from the medical records was tumbling (*n* = 6), traffic accident (*n* = 3), crashing (*n* = 5), and sports injury (*n* = 1). The average duration of nonunion before the index intervention was 126.8 ± 124.2 months (range, 17–368 months), and each patient had undergone at least one failed surgical fixation for the nonunion.

**Table 2:** Postoperative outcomes

Patient	Follow-up period, months	outcome	Duration of bone healing, months	Angulation	VAS (pre-pos)		Constant and Murley (pre-pos)		Mayo elbow performance index (pre-pos)		Complications
1	19	Union	5	<10°	5	0	74	96	65	100	None
2	45	Union	6	<10°	8	1	36	88	50	100	None
3	19	Union	6	<10°	4	0	69	84	75	100	None
4	16	Union	5	<10°	6	1	46	90	55	90	None
5	27	Union	7	<10°	7	2	32	91	45	85	Ulnar nerve palsy
6	14	Union	8	<10°	6	0	56	92	60	95	None
7	19	Union	10	>10°	5	1	36	78	55	85	Iliac crest discomfort
8	21	Union	7	<10°	6	2	34	68	45	80	None
9	31	Union	4	>10°	7	1	38	90	50	95	Superficial wound infection
10	32	Union	6	<10°	5	0	44	86	60	100	None
11	36	Union	4	<10°	4	0	72	94	70	95	None
12	15	Union	8	<10°	6	2	36	74	55	85	None
13	14	Union	5	<10°	7	3	55	78	50	85	Radial nerve palsy
14	24	Union	6	<10°	3	0	66	92	80	100	None
15	13	Union	9	<10°	5	0	63	90	60	95	None

**Fig. 3:**
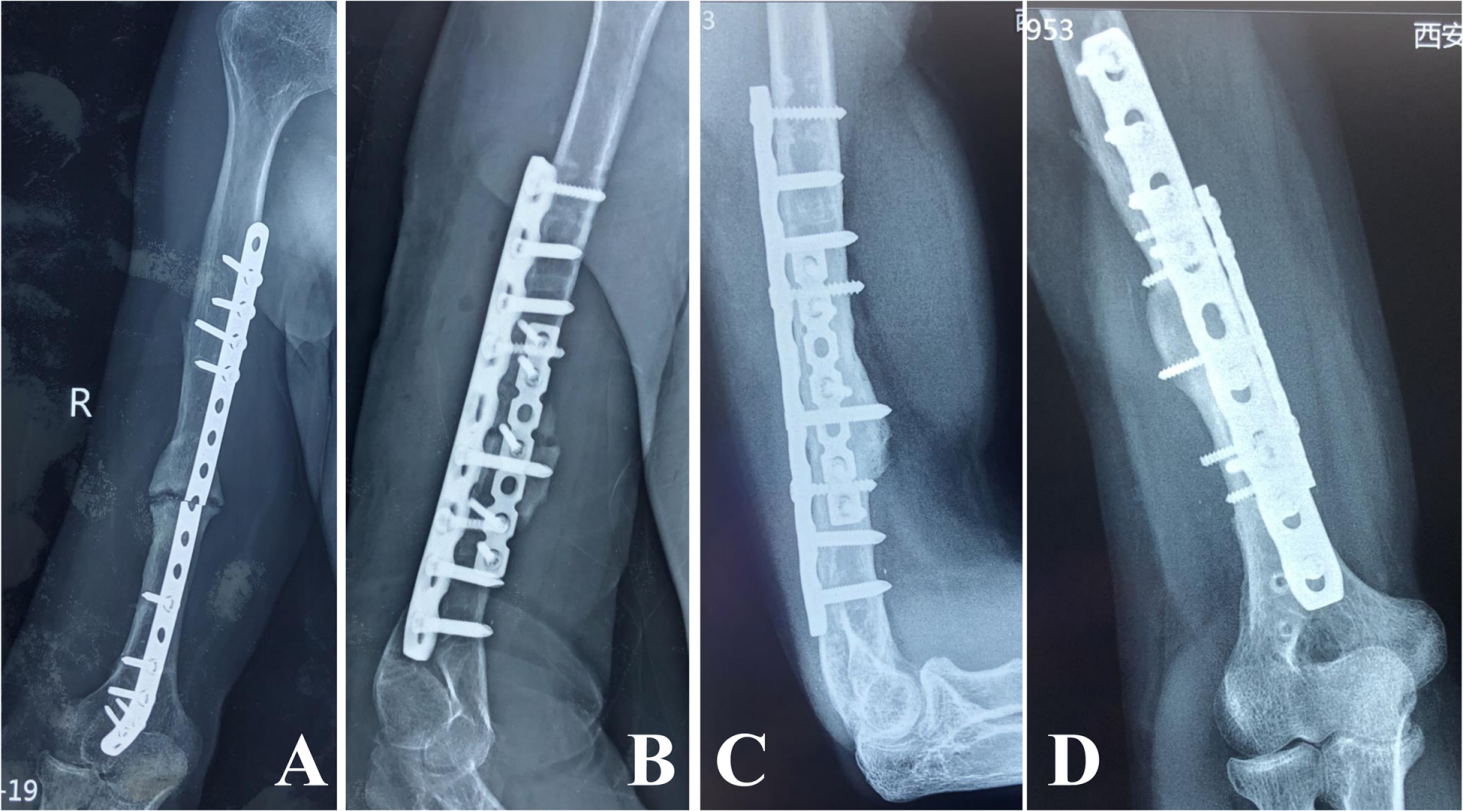
Images from a 42-year-old man with right humeral shaft nonunion for more than 26 years who had undergone four surgeries before seeking treatment at our institution. **a** Radiograph showing nonunion with implant failure at 4 years after the last revision. **b** Fixation using double locking compression plating and bone grafting. **c**, **d** Radiographs demonstrating osseous union at 8 months postoperatively

Patients were followed-up for an average of 23.0 ± 9.4 months (range, 13–45 months). Each fracture had solid clinical and radiographic evidence of union after 6.4 ± 1.8 months (range, 4–10 months), and none of the implants had loosening or breakage at final follow-up. The postoperative alignment was within 10° of anatomic alignment in 13 patients, while two patients had angulation of more than 10°. The mean Constant and Murley shoulder function score significantly improved from 50.5 ± 15.3 preoperatively (range, 32–74) to 86.1 ± 8.1 at final follow-up (range, 68–96) (*P* < 0.001), with the result classified as excellent in 11 patients and good in four. For the elbow, the mean MEPI significantly improved from 58.3 ± 10.5 preoperatively (range, 45–80) to 92.7 ± 7.0 at final follow-up (range, 80–100) (*P* < 0.001), with the result classified as excellent in 10 patients and good in five. The mean VAS score significantly decreased from 5.6 ± 1.4 preoperatively (range, 3–8) to 0.9 ± 1.0 at final follow-up (range, 0–3) (*P <* 0.001). All patients were able to resume work and were very satisfied with the treatment (Figs. 2, 3).

Complications were seen in four patients. One patient developed a superficial wound infection at the nonunion site, which resolved after 4 weeks of antibiotics and dressing changes. One patient developed radial nerve palsy and one developed ulnar never palsy; in both cases, the palsy manifested as persistent numbness in the fingers without movement dysfunction at final follow-up. One patient reported occasional discomfort in the bone graft donor area at the iliac crest.

## Discussion

Despite great advances in orthopedic technology, surgeons still encounter patients with humeral shaft nonunion who have already undergone repeated failed surgeries. In some circumstances, repeated operative failures to obtain union coupled with soft tissue maladaptation and deformity have left the patient with a profound disability and an abandonment of optimism, especially for patients with poor financial conditions [2, 3, 7, 10, 20]. Several methods have been designed to treat humeral shaft nonunion by providing adequate fixation across the fracture site and improving the local biomechanical environment or blood supply, but each method has its drawbacks. Well recognized revisions for humeral shaft nonunion include interlocking nail fixation, Ilizarov external fixation, and internal plate fixation with an autologous iliac crest bone graft or vascularized fibular graft; of these methods, plating with bone grafting is generally considered the first choice for nonunion of the humeral shaft [9, 10, 12].

Interlocking intramedullary nails have been widely used in acute humeral fractures, pathologic fractures, and nonunions of the tibia or femur shaft [3]. For humeral shaft nonunion, nailing or exchange nailing reportedly improves the biomechanical stability via the use of a nail at least 1 mm thicker than the shaft diameter and fosters a healing environment by transporting mesenchymal stem cells into the nonunion sites during the reaming procedure [2, 21]. However, the healing rate of nailing for humeral shaft nonunion varies from 40 to 95.6% [22–24]. A poor outcome might result from a lack of cyclical loading due to weight-bearing and a higher amount of distractive and torsional loads on the humerus [23]. As most of the patients in the present study had erosion, osteopenia, and sclerotic bone, it was difficult to achieve adequate fixation with good rotational control using exchange nailing; furthermore, as most patients had stiffness in the neighboring joints, exchanging nailing might have caused subacromial impingement and rotator cuff injury, which would have worsened the function of those joints [25]. Therefore, exchange nailing or nailing was not performed in the current study.

External fixation provides good stability and compression of the nonunion sites to achieve bony consolidation. Traditionally, Ilizarov ring fixators are used for distraction osteogenesis and bone transport in patients with infected nonunion of the tibia or femur. Several studies report that this technology yields a high union rate in patients with nonunion of the humeral shaft [26, 27]. However, the disadvantages of external fixation include a long fixation time, risk of pin-tract infection, and patient discomfort, making it an unreliable and unnecessarily complex option for non-infected nonunion [10].

Plating combined with bone grafting is the method most widely used to treat humeral shaft nonunion, as it achieves precise correction of the deformity and absolute stability, and enables the use of biologic augmentation [5, 13]. One study reported a healing rate of 97% for anterior augmentation plating of aseptic humeral shaft nonunion [28], and a review of 36 studies found that plating with autologous bone grafting achieves a union rate of up to 98% in patients with humeral shaft nonunion [29]. Furthermore, plate fixation and bone grafting is recommended for recalcitrant humeral shaft nonunion following initial operative fixation of the index fracture [30]. In the present study, double plate fixation combined with autologous iliac crest structural bone grafting achieved excellent or good outcomes in patients with recalcitrant humeral shaft nonunion after prior failed surgeries. This treatment might have the following advantages. Firstly, besides attaining precise deformity correction and absolute stability, fixing a second plate vertically to the anterior portion of the bone graft for structural support maintains intimate contact between the bone graft and both nonunion segments, maximizing the osteoconductive, osteogenic, and osteoinductive properties of autologous bone. Secondly, double-plate technology is very effective in restoring the intact compressive and torsional stiffness of humeral shaft segments [31, 32]; thus, postoperative functional rehabilitation was able to be started on the next postoperative day without the use of external immobilization, facilitating limb function restoration.

The plating technique also has complications, such as screw back-outs, peripheral nerve paralysis, and infection [31]. In the present study, there were two patients with nerve palsy (one with ulnar nerve palsy and one with radial nerve palsy) and one with a superficial wound infection. Fortunately, both patients with nerve palsy only had persistent finger numbness without movement dysfunction. For patients with abundant scar tissue from multiple surgeries, surgeons must protect the nerves as much as possible during neurolysis. No patients had implant failure at final follow-up.

Few studies have focused exclusively on the treatment of recalcitrant humeral shaft nonunion. Borus et al. [2] performed uniform surgical repair with 4.5-mm compression plating in combination with bone grafting in seven patients with humeral diaphyseal nonunion following at least two failed prior surgical procedures, and reported that all nonunions had healed with good function of the affected extremity at final follow-up. Marti et al. [8] reported a series of 51 patients with humeral shaft nonunion, 10 of whom had undergone at least two prior surgical procedures; all patients were treated with plating and autogenous bone grafting, and all achieved union at 1 year postoperatively, with 96% of patients achieving excellent or good function. Adani et al. [33] performed plate fixation and fibular transplantation in 13 patients with an average humeral defect length of 10.5 cm who had undergone at least two surgeries; nine patients healed primarily, three required additional bone grafting, and one required a second fibular transplant. In the current study, each patient had previously undergone at least one failed operation for nonunion before the index intervention procedure. Double plating and autologous bone grafting resulted in a bone healing rate of 100%, with a mean bone healing time of 6.4 ± 1.8 months. At final follow-up, each patient showed significantly improved function of the affected limb and significantly reduced pain. Thus, the outcomes of the present study are consistent with the above mentioned studies.

The limitations of the present study are related to its retrospective nature and small sample size. We were also unable to directly compare plate fixation with other fixation strategies. Finally, because of the rarity and complexity of this specific situation, it was not possible to include a control group. Despite these limitations, the present study demonstrates that double plating in combination with autologous bone grafting achieved successful outcomes for recalcitrant humeral shaft nonunion. To the best of our knowledge, this is the largest series of patients who had undergone multiple surgeries for nonunion and were then treated using double plating technology.

## Conclusion

Double LCP vertical fixation in combination with autologous iliac cancellous bone grafting provides absolute stable fixation of the nonunion segments, which enables the early initiation of functional exercise and the use of structural autologous bone grafts for optimal bone healing. The present technique is a valuable option for the treatment of recalcitrant humeral shaft nonunion that has failed prior surgical intervention.

## Data Availability

The datasets used/or analyzed during the current study are available from the corresponding author on a reasonable request.

## Abbreviations

LCP: Locking compression plate
MEPI: Mayo elbow performance index
VAS: Visual analog scale
ORIF: Open reduction and internal fixation
BMP: Biologic augmentation
CRP: C-reactive protein
ESR: Erythrocyte sedimentation rate
